# The association of low birth weight with serum C reactive protein in 3‐year‐old children living in Cuba: A population‐based prospective study

**DOI:** 10.1002/ajhb.22936

**Published:** 2016-11-18

**Authors:** Silvia Josefina Venero‐Fernández, Hermes Fundora‐Hernández, Lourdes Batista‐Gutierrez, Ramón Suárez‐Medina, Esperanza de la C. Mora‐Faife, Gladys García‐García, Ileana del Valle‐Infante, Liem Gómez‐Marrero, John Britton, Andrew W. Fogarty, HINASIC (Historia Natural de la Sibilancia en Cuba/Natural History of Wheezing in Cuba) Study Group

**Affiliations:** ^1^ Instituto Nacional de Higiene, Epidemiología y MicrobiologíaInfanta No 1158 e/ Llinás y ClavelCódigo Postal 10300La HabanaCuba; ^2^ Nottingham Biomedical Research Unit, Division of Epidemiology and Public HealthUniversity of Nottingham, Clinical Sciences Building, City HospitalNottinghamNG5 1PBUnited Kingdom

**Keywords:** birthweight, children, Cuba, inflammation

## Abstract

**Objective:**

Low birthweight is associated with a decreased risk of childhood leukemia and an increased risk of both cardiovascular disease and all‐cause mortality in adult life. Possible biological mediators include systemic innate immunity and inflammation. We tested the hypothesis that birthweight was inversely associated with serum high sensitivity C reactive protein assay (hsCRP), a measure of both innate immunity and systemic inflammation.

**Methods:**

Data on birthweight and current anthropometric measures along with a range of exposures were collected at 1 and 3 years of age in a population‐based cohort study of young children living in Havana, Cuba. A total of 986 children aged 3‐years‐old provided blood samples that were analyzed for serum hsCRP levels.

**Results:**

Nearly 49% of children had detectable hsCRP levels in their serum. Lower birthweight was linearly associated with the natural log of hsCRP levels (beta coefficient −0.70 mg L^−1^ per kg increase in birthweight, 95% CI: −1.34 to −0.06). This was attenuated but still present after adjustment for the child's sex and municipality (−0.65 mg L^−1^ per kg birthweight; 95% CI: −1.38 to +0.08). There were no associations between growth from birth or anthropometric measures at 3 years and systemic inflammation.

**Conclusions:**

Birthweight was inversely associated with serum hsCRP levels in children aged 3 years living in Cuba. These observations provide a potential mechanism that is present at the age of 3 years to explain the association between low birthweight and both decreased childhood leukemia and increased cardiovascular disease in adults.

## 1 Introduction

Exposures from conception onwards are well recognized to impact birthweight and subsequent diseases in adulthood (Gluckman et al., 2008). The associations between lower birthweight and decreased risk of childhood leukemia (Belson et al., 2007; Hjalgrim et al., 2004; Paltiel et al., 2004) and increased cardiovascular disease (Huxley et al., 2007; Mu et al., 2012; Risnes et al., 2011) and all‐cause mortality (Risnes et al., 2011) in adults are well recognized although the pathogenetic mechanisms are unclear. Potential biological processes that may be on the casual pathway for these observations include innate immunity whereby immune surveillance prevents the development of aberrant cells into clinical malignancies (Bindea et al., 2010; Chow et al., 2011; Ostrand‐Rosenberg, 2008), and systemic inflammation, which is known to be a risk factor for cardiovascular disease (Danesh et al., 2000; Ridker and Cook, 2004; Ridker et al., 1998, 2000). High sensitivity C reactive protein (hsCRP) is a member of the pentraxin protein family, and is a biomarker that contributes to both innate immunity (Du Clos and Mold, 2004; Simons et al., 2014) and also systemic inflammation (Black et al., 2004; Mortensen, 2001), and is positively associated with higher all‐cause mortality in adults (Zacho et al., 2010). Prospective studies in children may increase understanding of these associations by determining if birthweight is associated with increased hsCRP levels in later childhood.

Cuba has an excellent health care system that delivers infant mortality rates comparable to much richer countries (Cooper et al., 2006), with good health infrastructure providing a unique environment where risk factors for disease can be studied. We have used data from a prospective study in young children to test the primary hypothesis that low birth weight is associated with increased hsCRP levels at the age of 3 years.

## 2 Materials and methods

### 2.1 Study population and setting

The study design is a prospective observational epidemiological study. All children aged between 12 and 15 months who were living in Havana, Cuba, between March 2010 and March 2011 and who attended one of 17 policlinics, nested in four municipalities in Havana, Cuba, were eligible to be randomly selected to participate in the study (Arroyo Naranjo, Cerro, Habana del Este, La Lisa). Recruitment for the study (Appendix) has been described in detail elsewhere (Venero‐Fernandez et al., 2013). The study protocol was approved by the local Havana Scientific Committee in Cuba and also by the University of Nottingham Medical School Ethics Committee in the United Kingdom.

### 2.2 Data collection

The study was initially designed to identify environmental exposures that may increase the risk of asthma or allergic disease in Cuba (Fundora Hernández et al., 2014; Suarez‐Medina et al., 2014; Venero‐Fernandez et al., 2013). The baseline data collection in eligible children at the age of 1 year consisted of an interviewer‐administered questionnaire that collated the responses from the parent/caregiver about prenatal and postnatal exposures of the child, their living environment, and the medical history of the family. Birthweight was obtained from the child's medical records of the readings taken at the place of birth. Specific questions focused on smoking history, including that of the mother during pregnancy, and also information on exposure to individuals who smoke tobacco in the infant's home. The children and their caregivers were resurveyed 2 years later when the children were ∼3‐years‐old and clinically stable. Data on the height and weight at the time of the interview were also collected using the measuring equipment and weighting scales available in the policlinic which were calibrated every 6 months by the Ministry of Health. For those 3‐year‐old children whose parents gave consent, a blood sample was taken. This was frozen and subsequently analyzed for serum hsCRP using an immunoturbidimetric assay (SpinReact, Spain) on a SpinLab 100 (SpinReact, Spain) analyzer in a standardized manner designed to minimize measurement error (Spinreact high sensitivity CRP assay).

### 2.3 Data analysis

The data were entered into an electronic database, cleaned, and checked for errors. All statistical analyses were carried out in Stata v14 (StataCorp, Texas) using the survey commands (svyset) to allow for the clustered survey design (Stata Corp). The distribution of serum hsCRP levels was transformed into natural logarithms after adding +0.00001 to accommodate undetectable values.

Linear regression analyses were then performed using logCRP at the age of 3 years as the primary endpoint and birthweight as the main independent variable. The associations of weight at 3 years and change in weight since birth with logCRP were secondary endpoints. A number of further exposures were added to the model in a stepwise manner and they were retained if they changed the size of statistically significant associations by 10% or more. These were municipality, sex, duration of exclusive breastfeeding (categorical), mother smoking in pregnancy, presence of smokers in the household at 1 year and at 3 years of age (categorical), self‐reported pollution in their locality at age 1 year and at 3 years of age, mother's age (categorical), number of siblings (categorical) and maternal education (categorical). Interactions were explored for sex using the likelihood ratio testing with a *P* value of <0.10 as evidence of an interaction. To explore the possibility of subclinical infections driving higher levels of serum hsCRP, sensitivity analyses were implemented restricting the study population to those with an hsCRP level of less than either 10 or 20 mg L^−1^. Assumptions of linearity were tested by comparing the linear model with the categorical model using likelihood ratio tests.

## 3 Results

**Figure 1:**
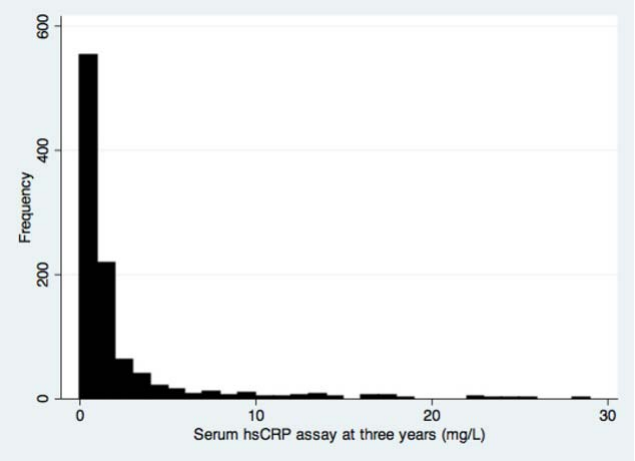
Distribution of serum hsCRP levels in study population

**Figure 2:**
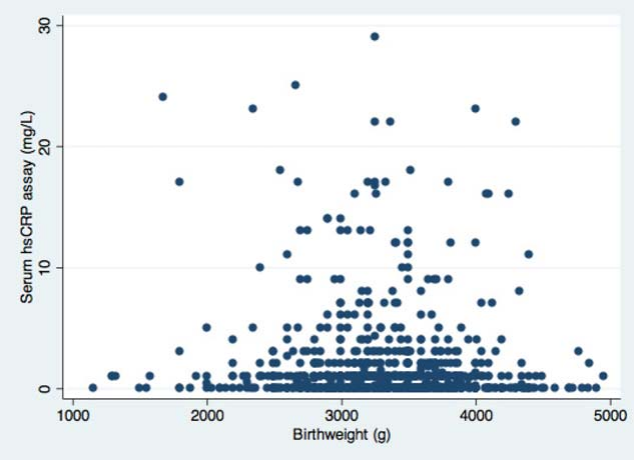
Scatter graph of the association between serum high sensitivity CRP levels and birthweight

Of the 3‐year‐old 1,543 children who participated in the survey, 986 (64%) provided blood samples for analysis of serum CRP levels. Children who provided blood were similar to those who did not in terms of birthweight, but were slightly more likely to live in the Habana del Este and Arroyo Naranjo municipalities than those who did not contribute blood samples (Table 1). Forty‐nine percent of children who provided blood samples had a detectable serum CRP level and the distribution is presented in Figure 1, with a lowest detectable value of 0.01 mg L^−1^. The association with birthweight presented in Figure 2.

**Table 1:** Characteristics of study participants

Variable	Definition of category	All children: *N* = 1543	Children who did not give blood *N* = 557	Children who gave blood *N* = 986
Mean age in years (range)		3 (3–3)	3 (3–3)	3 (3–3)
Skin color (%)	White	695 (45)	256 (46)	439 (45)
	Mixed	652 (42)	238 (43)	414 (42)
	Black	196 (13)	63 (11)	133 (13)
Gender (%)	Female	753 (49)	278 (50)	475 (48)
	Male	790 (51)	279 (50)	511 (52)
Municipality (%)	Habana Este	480 (31)	135 (24)	345 (35)
	Cerro	229 (15)	88 (16)	141 (14)
	La Lisa	264 (17)	204 (37)	60 (6)
	Arroyo Naranjo	570 (37)	130 (23)	440 (45)
Median number of siblings (IQR)	–	1 (0–2)	1 (0–1)	1 (0–2)
Median duration of breastfeeding, months, (IQR)		4 (2–5)	4 (2–5)	4 (2–5)
Mean age of mother at birth in years (sd)	–	26.7 (6.2)	26.2 (6.2)	26.9 (6.2)
		*N* = 1542		*N* = 985
Mothers education	Primary	26 (2)	11 (2)	15 (2)
	Secondary	417 (27)	162 (29)	255 (26)
	Pre‐University	895 (58)	305 (55)	590 (60)
	University	205 (13)	79 (14)	126 (13)
Smokers living in home at 1 year	No	726 (47)	258 (46)	468 (47)
	Yes	817 (53)	299 (54)	518 (53)
Smokers living in home at 3 years	No	742 (48)	267 (48)	475 (48)
	Yes	801 (52)	290 (52)	511 (52)
Self‐reported pollution at home at 1 year	Yes	1126 (73)	423 (76)	703 (71)
	No	417 (27)	134 (24)	283 (29)
Self‐reported pollution at home in 3 years	Yes	1058 (69)	376 (68)	682 (69)
	No	485 (31)	181 (32)	304 (31)
Birthweight, kg (sd)	–	3.32 (0.52)	3.31 (0.52)	3.33 (0.52)
		*N* = 1540	*N* = 556	*N* = 984
Weight at 3 years, kg (sd)	–	15.83 (2.58)	15.66 (2.62)	15.93 (2.55)
Height at year 3, m (sd)	–	0.96 (0.07)	0.95 (0.07)	0.96 (0.07)

IQR = interquartile range.

In the primary analysis of the association between birthweight and log serum hsCRP levels at age 3 years (Table 2), there was a significant linear association in the univariate analysis (−0.70 mg L^−1^ kg^−1^ birthweight, 95% confidence intervals CI: −1.34 to −0.06). There was no effect modification of this association by sex. These associations persisted in sensitivity analyses restricting the population to those with serum hsCRP measurements <20 mg L^−1^ (−0.65 mg L^−1^ kg^−1^; 95% CI: −1.37 to +0.78) and < 10 mg L^−1^ (−0.59 mg L^−1^ kg^−1^; 95%CI: −1.31 to +0.14).

**Table 2:** Association between anthropometric measures and systemic inflammation (log hsCRP) at 3 years

Weight measure	Univariate analysis	Model adjusting for municipality	Model adjusting for sex	Model adjusting for municipality and sex
Birthweight (kg)	−0.70 (−1.34 to −0.06)	−0.78 (−1.45 to −0.10)	−0.58 (−1.29 to +0.13)	−0.65 (−1.38 to +0.08)
Weight at 3 years (kg)	−0.05 (−0.22 to +0.13)			−
Change in weight in first 3 years (kg)	−0.02 (−0.22 to +0.19)			−

Beta‐coefficient = natural log CRP (mg L^−1^) per kg increase in birthweight.

Both place of location (municipality) and the child's sex were significant confounding factors (Table 2). Adding both of these variables to the model gave a similar association as seen in the univariate model (−0.65 mg L^−1^ kg^−1^; 95% CI: −1.38 to +0.08, *P* = 0.07). Repeating the univariate analysis with no adjustment for sampling gave a similar qualitative association with wider confidence intervals between the highest category of serum hsCRP levels and birthweight (−0.70 mg L^−1^ kg^−1^, 95%CI: −1.56 to +0.16).

There were no significant associations between CRP levels at the age of 3 years and either weight at 3 years, body mass index at 3 years, height at 3 years or change in weight at 3 years from birth. Similarly, there was no association with either the presence of smokers in the house at age 3 years or the duration of exclusive breastfeeding with logCRP levels.

## 4 Discussion

To our knowledge this is the largest prospective population‐based study to investigate the association between birthweight and serum hsCRP levels in 3‐year‐old children. Our data demonstrate that birthweight is inversely associated with serum hsCRP measurements at 3 years, with a one kg increase in weight associated with an ∼50% decrease in serum hsCRP. These associations have potential implications for understanding how low birthweight is associated with disease in later life.

The strengths of our data include the randomly selected prospective population‐based study from which these children were recruited and the detailed phenotypic information collected on a number of environmental exposures from pregnancy onwards, allowing consideration of potential confounding or causal exposures. The data on birthweight were collected from medical records obtained as part of the infant's postnatal care, giving confidence that these data are not influenced by recall bias. Subsequent data on weight and height at the age of 3 years were also collected at the policlinic using validated medical equipment. The blood samples were collected, processed and stored in a standardized manner and then analyzed in batches in a central laboratory, providing confidence in the integrity of these data. The persistence of the associations after sensitivity analyses restricted to lower levels of serum hsCRP suggests that these observations are robust.

Although we were only able to collect blood samples for analysis from 64% of those who attended, significant selection bias is unlikely as those who did provide blood were generally similar to those who did not. The blood samples were collected in the early morning through the year in a tropical country and hence temperature and ambient pollution may be expected to vary according to season and weather conditions. We are unable to adjust for these factors, but anticipate that they are most likely to generate a non‐systemic measurement error in CRP values that would not bias our observations. Similarly, although we only recruited individuals with no current symptoms of infection, we were unable to exclude infection at the time of blood collection; again this would be expected to lead to non‐systemic measurement error, and hence would be unlikely to bias our observations unless low birthweight increased susceptibility to infection and hence prevalence of subclinical infection.

There are no existing population‐based studies of the association between birthweight and serum hsCRP levels in young children that we are aware of. One study from the UK in 553 children aged 10–11 years reported a median hsCRP value of 0.15 mg L^−1^ and no association with birthweight either before or after adjustment for current body size (Cook et al., 2000). An analysis of 996 Mexican American children aged 6–11 years who provided data for the Third National Health and Nutrition Survey in 1988–1994 in the USA reported that 11% of children had CRP values of 2.1 mg L^−1^ or more, and that again there was no association with birthweight in this population (Gillum, 2003). One limitation of the second study is the unavailability of the high sensitivity CRP assay that precludes direct comparison with our study population. However, the aforementioned two studies in children aged 6–11 years did report a positive association between body habitus and serum CRP, which was not present in our larger dataset. It is possible that the mechanism that drives the well recognized association between adiposity and serum hsCRP (Fogarty et al., 2008) is not established at the age of 3 years.

Other studies of the association between birthweight and CRP levels have been in adults and these studies are probably more susceptible to confounding by environmental factors as the participants will have had more time to accumulate exposures. In particular, it is unclear if current body fat is on the causal pathway or may be considered a confounding factor. The potential importance of confounding by adiposity is reflected in the fact that the majority of studies of the association between birthweight and CRP in adults only observed an association after adjustment for body habitus. The MIDSPAN family study reported no initial association between serum hsCRP and birth weight in preliminary analyses in 1663 individuals aged 30–59 years, but after adjustment for body mass index an inverse linear association between serum hsCRP and birth weight was observed, with a 10.7% decrease in hsCRP for each 1kg increase in birth weight (Sattar et al., 2004). Data from the Bogalusa Heart Study similarly reported an inverse linear association between hsCRP in 776 adults aged 24–43 years after adjusting for the confounding factors that included BMI (Bhuiyan et al., 2011). Data from 1461 participants from a birth cohort in the Philippines who were aged 21 years found no initial association between birthweight and serum CRP in tertiles in initial analyses, but a negative association was observed after adjustment for a number of confounding factors including current waist circumference and skinfold thickness (McDade et al., 2010). Data from a birth cohort from Finland of 5840 participants aged 31 years, observed that hsCRP levels were 16% higher for each 1 kg decrease in birth weight after adjustment for body mass index, and 6% higher without adjustment for BMI (Tzoulaki et al., 2008). Analysis of data from the US National Longitudinal Study of Adolescent Health reported that, at the age of 24–32 years, the association between birth weight and serum hsCRP was non‐linear, being positive for birth weights <2.8 kg and above this threshold the association was negative (McDade et al., 2014).

The observation that low birthweight is associated with higher levels of cardiovascular disease (Huxley et al., 2007; Mu et al., 2012; Risnes et al., 2011) and all‐cause mortality in adults is well recognized. The association of higher birthweight with childhood leukemia also has been the topic of a number of studies (Belson et al., 2007; Hjalgrim et al., 2004; Paltiel et al., 2004). Our observations that low birthweight is associated with higher circulating hsCRP levels at the age of 3 years do not demonstrate that this is the biological mechanism that explains these observations. However, it does permit us to speculate that via its role in contributing to innate immunity (Du Clos and Mold, 2004; Simons et al., 2014), inflammation may play a role in the subsequent susceptibility to the development of diseases seen in individuals with low birthweight (Black et al., 2004; Mortensen, 2001). There is no clear direction of causality as children with low birth weight have more respiratory infections compared to a control population (Chan et al, 1989), while it is recognized that CRP boosts the innate immune response to bacteria (Ng et al., 2007), with particular activity against *Streptococcus* pneumonia (Thomas‐Rudolph et al., 2007). Further longitudinal studies of the association between birthweight and diseases will be required to clarify these issues, adjusting for serum hsCRP levels in childhood.

In summary, we have identified that in a population of children aged 3 years, there is an inverse association between birthweight and serum hsCRP levels. These data provide one potential explanation for how low birthweight is associated with changes in risk of disease and mortality in later life. Further follow up of this cohort with the particular addition of cardiovascular outcome measures will permit increased understanding of the biological processes that drive these conditions.

### National Institute of Hygiene, Epidemiology and Microbiology

Silvia J Venero‐Fernández, Ramón Suárez‐Medina, Hermes Fundora‐Hernández, Lenina Menocal‐Heredia, Yuria Isabel Caraballo‐Sánchez, Félix Manuel Rosado‐García, Reina Amelia Quintana, Lourdes Batista Gutiérrez. Patricia Varona‐Pérez, María del Carmen Hinojosa.

### Hospital Universitario Pediátrico Docente Centro Habana

Regla N. Rivero, Javier Muñoz‐Pérez, Caridad González‐Morfa.

### Municipio de Arroyo Naranjo

Esperanza de la C. Mora‐Faife, Damaris Zaldívar‐Ricardo, María Teresa Méndez‐Ratger, Amed Fernández‐Casamayor, Gisela Álvarez‐Valdez, Anadelis Alfonso‐Hernández, Roberto Hidalgo‐Mederos, Nereida Calderin‐Martínez, .Jorge Antonio Febles‐del Toro, Danay Silva, Violeta Suarez‐Angelo, Noelvis Zayas‐Mompie, Grisel M. Esquivel‐Barrios, Zoe de los Angeles Figueroa‐Barreto, Olga Lidia Negrin‐Molina, Odalis Kessell‐ Díaz, Mariela de la Caridad Hernández‐González, Dulcima Casanave‐Guarnaluce, Gretel Comas‐Fonseca, Vilma Álvarez‐Valdez, María Engracia‐Báez Rodríguez, Felicia Sánchez‐Cardentey, Nieves Sardinas‐Báez, Roberto Esteban Márquez‐Solís, Marlene Flores‐Carballosa, Nagaby Gómez‐ Baro.

### Municipio de La Lisa

Gladys García‐García, María de Lourdes Ortiz‐Hernández, María Antonia Betancourt‐López, Marlén Batista‐Cedeño, Iris Alfonso‐Castellanos, Leticia Gómez‐García, Ernesto Rafael Gutiérrez‐Mendoza, María Luisa Loynaz‐González, Nibenia Rodríguez‐Trujillo, Yanet Pozo‐Herrera, Víctor Manuel Montejo‐Guerra, Julia Urbina‐Reynaldo, Valentina Gómez‐Suliman, Caridad Alicia Rodríguez‐Aragón.

### Municipio de Cerro

Ileana del Valle‐Infante, Claudia Matos‐Ramos, Martha Betancourt‐Orue, Oscar Alba‐Monteagudo, Yuderkis Ferrer‐Ceruto, Aída Damas‐Martínez, Mercedes Peñalver‐Pérez

### Municipio de Habana del Este

Liem Gómez‐Marrero, Sarahí Castillo‐Martínez, Amor de los Angeles Castaño‐Vega, Norberto Torriente‐Barzaga, Ileana Ávila‐Rodríguez, Magalys Navarro‐Ruiz, Kirenia Díaz‐Hernández, Iluska De La Torre Suarez, Gilberto Roque‐Pereira, Yamilet Corona‐Carnero, Idania Gonzalaz‐Fernandez, Fidelia Romeu‐Ravelo, Regla Hernandez‐Ponce, Teresa Serrano‐González, Dulce Romeo‐Cepero, Caridad Gonzalez Leiva, Teresa De Jesus Cobas Espino, Nuris‐Fajardo, Midiala Perez‐Arcia, Sarahy Diaz‐Araujo, Yanet Medina‐ Lescay, Sandra Collazo‐Rodriguez, Julia Amparo Griñán‐Ramos, Teresa Serrano‐Gonzalez, Beatriz Lazo‐Vazquez, Tania Pupo‐Portal, Nidia Leyva‐Porra, Odalys Pacheco‐Mesa, Martha Rizo‐Ramos, Yaneysi Vallafuerte‐Perez, Aliniuska De La Paz‐Arias, Maite B Garcia‐Sotolongo, Yusimí Calzado‐Herrera, Martha Nidia Rizo‐Ramos, Guillermo Verdecia, Mayté B García‐Sotolongo, Juana F Abreu‐Quijano, Fidelia Romeo‐Ravelo.

Thanks also to all Municipality Directors and the laboratory workers who have also supported the study.
